# Diagnosis of three cases of endobronchial tuberculosis presenting as unresolved pneumonia, following fiberoptic bronchoscopic biopsy

**DOI:** 10.4103/0970-2113.68316

**Published:** 2010

**Authors:** Partha Pratim Roy, Subir Kumar Dey, Anirban Sarkar, Amiya Kumar Dwari, Ankan Banerjee, Rik Banerjee

**Affiliations:** *Department of Pulmonary Medicine, Medical College, Kolkata, India*

**Keywords:** Endobronchial tuberculosis, fibreoptic bronchoscopy, unresolved pneumonia

## Abstract

Nowadays, endobronchial tuberculosis is of rare occurrence. This article presents three such cases. All of them presented as unresolved pneumonia with collapse-consolidation in chest X-ray. All the three patients were sputum smear negative for acid fast bacilli. Diagnosis was possible only with fiberoptic bronchoscopy and bronchial biopsy.

## INTRODUCTION

Unresolved pneumonia (UP) is a common problem encountered in day to day practice. Common causes are aspiration of foreign bodies, space occupying lesions (SOL) in lung, bronchiectasis, sequestration lung, etc. Rarely, endobronchial tuberculosis (EBTB) may present as unresolved pneumonia. In this report, three cases of endobronchial tuberculosis with unresolved pneumonia, who were diagnosed following fiberoptic bronchoscopy (FOB) and biopsy, are presented. In all the three cases of EBTB, sputum for AFB was negative.[1,2]

## CASE REPORTS

### Case 1

#### Presentation

A 19-year-old, unmarried female named Ms. PM (nonalcoholic, nonsmoker, nondiabetic and normotensive) hailing from a rural area of state West Bengal was admitted to our inpatient department with complaints of dry cough for two and half months along with fever and shortness of breath for one and half months duration.

Cough was non productive with increased severity at night and not associated with hemoptysis. Fever was of low grade, not associated with chills and rigors, headache or joint pains and was relieved by taking antipyretics. Shortness of breath was progressive in nature, aggravated by strenuous activities and subsided on taking rest. The patient also complained generalized weakness and significant weight loss over last 2 months. There was no history of convulsions or foreign body aspiration. Menstrual history was normal.

Before admission, she had taken several oral antibiotics. However, there was no history of taking ATD. None of her family members were documented to have suffered from TB.

#### Examination

She was thinly built and nutritional status was poor. There was significant pallor but no cyanosis, jaundice, clubbing or peripheral lymphadenopathy. She was afebrile and her resting respiratory rate was about 20/min. On examination of respiratory system, movement and vocal fremitus were decreased in Right interscapular, infrascapular and infra-axillary regions with impaired percussion note in the same. Auscultation revealed diminished vesicular breath sound and vocal resonance in the above-mentioned areas along with few crackles in the right interscapular and infrascapular regions. CVS and abdominal examinations were normal.

#### Investigations

**Figure 1:**
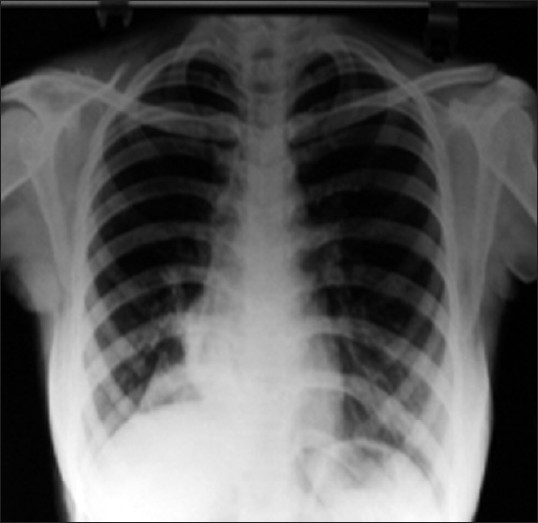
Chest X-ray (PA view) showing segmental collapse of right lower lobe

**Figure 2:**
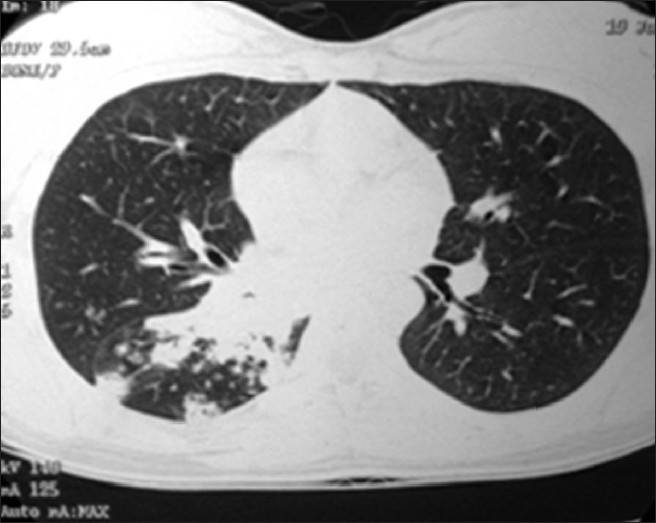
HRCT thorax showing segmental consolidation with treein-bud appearance

Complete hemogram revealed anemia (Hb: 9.7 gm %), neutrophilic leukocytosis (TLC: 12,000/mm^3^, DLC: N _70_) and high ESR (40 mm/h). Blood biochemistry was normal and HIV serology was nonreactive. Her sputum smear failed to show any AFB on two occasions. Chest X-ray revealed segmental collapse-consolidation of right lower lobe [Figure 1]. CECT thorax showed segmental consolidation with centrilobular nodules and tree-in-bud appearance [Figure 2]. Fiberoptic bronchoscopy revealed whole of right lower lobe bronchus filled with white gelatinous material obstructing bronchial lumen. Biopsy was taken and sent for HPE. BAL fluid was sent for Gram stain and C/S, AFB stain and M. Tuberculosis culture. While BAL fluid failed to show any organism in smear, the bronchial biopsy showed typical caseating epitheloid granuloma. Postbronchoscopic sputum did show AFB in smear examination.

#### Treatment

Initially, the patient was put on intravenous antibiotics. After review of investigation reports, antibiotics were omitted and Category I ATD (2H_3_R_3_Z_3_E_3_/ 4H_3_R_3_) started on 02.08.2008 (DOTS) along with oral prednisolone at a dose of 1.5 mg/kg body weight, gradually tapered over 8 weeks. Patient showed marked improvement both clinically and radiologically following treatment with anti-tubercular drugs.

### Case 2

#### Presentation

A 52-year-old gentleman, owner of a laundry business in the suburbs, (nonalcoholic, and nonsmoker, nondiabetic and normotensive) presented to our outpatient department with chief complaints of cough with expectoration, recurrent streaky hemoptysis and occasional low grade fever for last one and half years.

The expectoration was mucoid, scanty in amount and there was no postural or seasonal variation. Fever was of low grade (up to 100°F), and irregular, i.e. without any definite periodicity. There was no history of weight loss and appetite was well preserved.

He denied any history of contact with tuberculosis patient or any past history of ATD intake.

#### Examination

The patient was well built and well nourished. There was no clubbing or peripheral lymphadenopathy. His vitals were normal. Respiratory system examination was essentially normal except impaired percussion note and diminished vesicular breath sound in the left infraclavicular region. Trachea was central in position. CVS and CNS and abdominal examinations were unremarkable.

#### Investigations

**Figure 3:**
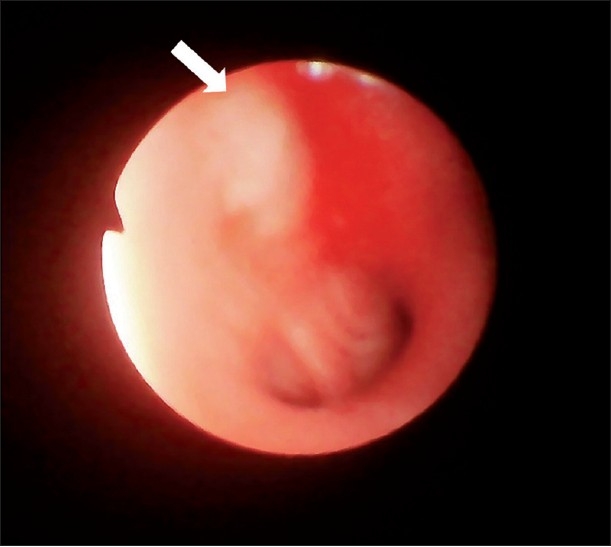
Caseating mass occluding upper division of left upper lobe bronchus with inflamed mucosa

Complete hemogram revealed anemia (Hb: 10.8 gm %) and high ESR (40 mm/hr). Blood biochemistry was normal and HIV serology was nonreactive. Her sputum smear failed to show any AFB on two occasions. Chest X-ray revealed nonhomogenous opacity in left upper zone with upward and forward retraction of oblique fissure, left hilar prominence and raised left hemidiaphragm. CECT thorax showed collapse-consolidation with few air bronchogram and tiny calcification in the left upper lobe. Fiberoptic bronchoscopy revealed hyperemic and swollen bronchial mucosa of left upper lobe. A globular mass was seen, partially occluding the left upper lobe opening [Figure 3]. After taking biopsy from the mass, white cheesy material was seen coming out of it. Biopsy was sent for HPE and BAL fluid sent for routine analysis, AFB stain and MTB Culture. Biopsy was taken and sent for HPE. BAL fluid was sent for Gram stain and C/S, AFB stain and M. Tuberculosis culture. While BAL fluid failed to show any organism in smear, the bronchial biopsy showed typical caseating epitheloid granuloma. Postbronchoscopic sputum did show AFB in smear examination. The BAL fluid grew Mycobacterium tuberculosis after three weeks.

#### Treatment

After review of investigation reports, Category I ATD (2H _3_R_3_Z_3_E_3_/4H_3_R_3_) started under (DOTS) along with oral prednisolone according to the dose schedule mentioned in the case report 1. Three months post treatment, repeat bronchoscopy showed restored patency of left upper lobe bronchus with stenosis.

### Case 3

#### Presentation

A 56-year-old widow, housewife from Kolkata, Mrs. EB, presented to our O.P.D on 09.10.2008 with complaints of cough for one and a half years.

Cough was insidious in onset, progressive in nature and associated with expectoration of about half a cup per day. Sputum was white in color without any foul smell. There was no fever, chest pain, shortness of breath, wheezing or history of aspiration of foreign body.

She denied any past history of tuberculosis. None of her family members were known to have been affected either.

Prior to admission, patient had been treated by local physicians and in this period her sputum smear for AFB was negative on repeated occasions.

#### Examination

The patient was normotensive, had slight pallor without any cyanosis, clubbing or peripheral lymphadenopathy. On examination of respiratory system, trachea was central; percussion note was impaired in the right infrascapular region. There was bronchial breath sound and whisperingpectoriloquy without any crackles in the same region. CVS, CNS and abdominal examinations revealed no abnormality.

#### Investigations

**Figure 4:**
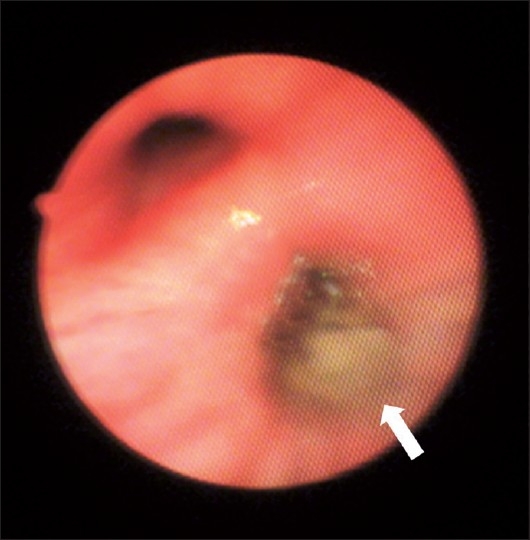
Pus at the opening of right lower lobe bronchus

Complete hemogram showed eosinophilia (15-18%) for last 5-6 months. Sputum smear was negative for AFB for three consecutive days. Repeated stool examination did not show any ova, parasite or cyst. HIV serology was nonreactive. Serum biochemistry reports were within normal limit. Chest skiagram and CT scan thorax revealed almost complete right lower lobe collapse-consolidation. During fiberoptic bronchoscopy frank pus was noted at the opening of right lower lobe bronchus, filling up the lumen [Figure 4]. BAL fluid sent for routine analysis and AFB smear, which was negative. Endobronchial biopsy was taken and sent for HPE, which established the diagnosis of tuberculosis.

#### Treatment

After review of investigation reports, Category I ATD (2H_3_R_3_Z_3_E_3_/4H_3_R_3_) started (DOTS) from 25.10.2008 along with oral prednisolone according to the dose schedule mentioned in the case report 1. At the end of 2 months, bronchial breath sound almost disappeared, radiological improvement was observed and continuation phase was started.

## DISCUSSION

Endobronchial tuberculosis is a relatively uncommon detected manifestation of a common disease like tuberculosis.[3] High index of suspicion is necessary when making the diagnosis in sputum negative patients.

Among the three cases of EBTB, one was a young female, the other two were middle aged, one female (56 years) one male (51 years).[4,5] Ip MS *et al* and Lee JH *et al* reported cases of EBTB in the young, with female predominance. Brande PM Vanden *et al* also reported that 15% of geriatric patients may have EBTB.[6]

Fiberoptic bronchoscopic appearance varied from granular gelatinous white in one, ulcerative in other, and tumor like with stenosis in third. Chung HS *et al* described seven subtypes depending upon FOB appearance- Actively caseating, ulcerative, hyperemic, granular, tumor like, non specific, tumor like.[7]

All the cases were diagnosed by FOB biopsy, though sputum for AFB was negative.[7] Chung HS *et al*. also reported high positivity of bronchoscopic biopsy (35% - 84%) and bronchial brushing 84.88%; Yield of BAL fluid analysis is lower than biopsy and brushing.

All three patients in our series responded well both clinically and radiologically to anti TB drugs and steroids. The dose of prednisolone was increased to 1.5 mg/kg to counterbalance the drug interaction between steroid and rifampicin.
